# Sequence polymorphism can produce serious artefacts in real-time PCR assays: hard lessons from Pacific oysters

**DOI:** 10.1186/1471-2164-9-234

**Published:** 2008-05-20

**Authors:** Nicolas Taris, Robert P Lang, Mark D Camara

**Affiliations:** 1 USDA- Agricultural Research Service, Hatfield Marine Science Center 2030 SE Marine Science Dr. Newport, OR 97365, USA; 2 Hatfield Marine Science Center, Oregon State University, 2030 SE Marine Science Drive, Newport, OR 97365, USA

## Abstract

**Background:**

Since it was first described in the mid-1990s, quantitative real time PCR (Q-PCR) has been widely used in many fields of biomedical research and molecular diagnostics. This method is routinely used to validate whole transcriptome analyses such as DNA microarrays, suppressive subtractive hybridization (SSH) or differential display techniques such as cDNA-AFLP (Amplification Fragment Length Polymorphism). Despite efforts to optimize the methodology, misleading results are still possible, even when standard optimization approaches are followed.

**Results:**

As part of a larger project aimed at elucidating transcriptome-level responses of Pacific oysters (*Crassostrea gigas*) to various environmental stressors, we used microarrays and cDNA-AFLP to identify Expressed Sequence Tag (EST) fragments that are differentially expressed in response to bacterial challenge in two heat shock tolerant and two heat shock sensitive full-sib oyster families. We then designed primers for these differentially expressed ESTs in order to validate the results using Q-PCR. For two of these ESTs we tested fourteen primer pairs each and using standard optimization methods (i.e. melt-curve analysis to ensure amplification of a single product), determined that of the fourteen primer pairs tested, six and nine pairs respectively amplified a single product and were thus acceptable for further testing. However, when we used these primers, we obtained different statistical outcomes among primer pairs, raising unexpected but serious questions about their reliability. We hypothesize that as a consequence of high levels of sequence polymorphism in Pacific oysters, Q-PCR amplification is sub-optimal in some individuals because sequence variants in priming sites results in poor primer binding and amplification in some individuals. This issue is similar to the high frequency of null alleles observed for microsatellite markers in Pacific oysters.

**Conclusion:**

This study highlights potential difficulties for using Q-PCR as a validation tool for transcriptome analysis in the presence of sequence polymorphism and emphasizes the need for extreme caution and thorough primer testing when assaying genetically diverse biological materials such as Pacific oysters. Our findings suggest that melt-curve analysis alone may not be sufficient as a mean of identifying acceptable Q-PCR primers. Minimally, testing numerous primer pairs seems to be necessary to avoid false conclusions from flawed Q-PCR assays for which sequence variation among individuals produces artifactual and unreliable quantitative results.

## Background

During the last decade, quantitative real time PCR (Q-PCR) has been widely employed in many fields of biological research (medicine, biotechnology, microbiology) and is considered to be the most sensitive and reliable method of quantifying mRNA transcripts [1]. In contrast to more traditional methods using image analysis to measure band intensity on gels and thus quantify PCR products at the final phase of the reaction, real time PCR exploits the kinetics of the PCR reaction [2], specifically the exponential phase of amplification during which the amount of the PCR product is theoretically proportional to the initial quantity of template [3]. Fluorescent reporter dyes and/or gene-specific probes allow for the detection and quantification of cDNA amplicons produced during each Q-PCR cycle. By either assuming perfect amplification efficiency in the reaction, or alternatively estimating amplification efficiency empirically from the data, it is possible to estimate with accuracy the concentration of the targeted nucleic acid sequence in the initial sample.

As Q-PCR technology has evolved and its use expanded, diverse protocols using chemistry ranging from non-specific reporter dyes to sequence specific probes and diverse instrumentation have been developed [4,5]. The specific chemistry and quality of the reaction components play an important role in optimizing Q-PCR reactions, underlining the requirement for critical evaluation in order to overcome subjectivity inherent to the Q-PCR assay [6]. As a consequence, Q-PCR can be a somewhat "fragile" assay because its accuracy depends on numerous factors such as template preparation [7], reagents [8,9], operator influence [8] and the mathematical/statistical validation procedure(s) used [10,11]. Furthermore, due to the exponential nature of the signal and typically the reduction of the kinetics of the signal to a single number (C_T_, the cycle number when sample fluorescence exceeds a chosen threshold above background fluorescence) which is used as an exponent in the estimation procedure, rigorous optimization of Q-PCR assays is especially critical. Even seemingly minor errors and artefacts are greatly magnified by exponentiation.

Numerous studies have examined the potential problems and pitfalls of Q-PCR assays [6,8,12], however, the influence of the primer (or probe) design on the accuracy of the assay has been directly addressed only rarely. While it is known that regions of low-complexity sequence can create problems for designing primer and probe sequences specific to the target sequence [13], the influence of polymorphism within the targeted sequence has received little attention even though this is particularly important when Q-PCR is used to complement and validate whole transcriptome analyses, such as differential display, suppressive subtractive hybridization (SSH) or cDNA-AFLP (complementary DNA Amplification Fragment Length Polymorphism). In these applications, Q-PCR assays generally target relatively short sequences, ranging from approximately 100 to 800 bp. In some cases, template sequence discrepancies or inaccuracies can lead to failed assays caused by poor or no binding of primers and probes and/or non-specific binding resulting in multiple PCR products. It is therefore critically important to verify the targeted sequence and to check for the presence of polymorphisms in the biological material under study. Unfortunately, one of the attractions of whole transcriptome analyses such as SSH or cDNA-AFLP is that they are designed for genome-wide expression analysis with no prior sequence information required, making this step difficult or even impossible in non-model organisms. Furthermore, even though DNA microarrays normally use known EST sequences, typically only in model organisms is sufficient sequence information available to examine levels of polymorphism although this is rapidly improving as more sequence information becomes available for non-model organisms.

In this study, we report on how sequence polymorphism impacts Q-PCR assays based on cDNA-AFLP analyses of mRNA transcription in *Crassostrea gigas*, a marine bivalve known for its high level of genetic variability [14,15]. Unlike SSH, cDNA-AFLP can be used directly for quantitative detection because the intensity of each fragment on a gel theoretically reflects the expression level of the gene [16]. However, Q-PCR is a valuable method to support the trends observed with cDNA-AFLP, especially since false positives are likely to occur using cDNA-AFLP.

We evaluated the expression of one EST [GenBank: EX956386] taken from a cDNA-AFLP library (Taris, unpublished data), and one EST taken directly from Genbank [GenBank: AJ565694]. We used Q-PCR to quantify the expression levels of these two ESTs. We designed and evaluated 14 primer pairs for each EST sequence and then used 6 and 9 primer pairs respectively that melt curve analysis indicated were suitable for Q-PCR analysis. Results are discussed in light of the impacts of sequence polymorphism on the results of Q-PCR quantification assays.

## Methods

### Biological material

We exposed fifty individuals from each full-sib family from a 50-family cohort of full-sib Pacific oysters to heat shock (43°C, 1 h) and subsequent starvation at ambient temperature and monitored their survival for 8 days post heat shock during November 2005. Based upon the percentage that survived following this stress challenge, we classified the families as either high surviving (H) or low surviving (L). We then chose four of the most extreme families (two with high and two with low survival) for further study. Sibs of the tested animals from these extreme families were over-wintered in flow-through seawater troughs to minimize the effect of estuarine environment on stress responses, and transcriptome analyses were conducted in summer 2006.

### Experimental design

Heat shock consisted of immersing twelve two-year-old oysters from each of the four families in sea water at 40°C for 1 h. Oysters were then returned to 17°C sea water in flow-through tanks. We collected gill tissue 6 h after the shock from six randomly chosen oysters per family.

### RNA extraction

We extracted total RNA from gill tissue using the RNeasy Mini Kit (QIAGEN) according to the manufacturer's instructions. Pieces of gill (~30 mg) were excised, and disrupted in 700 μl of RLT buffer (QIAGEN). Samples were treated with DNAse I (QIAGEN, RNase-Free DNase Set). We quantified RNA by measuring absorbance using a NanoDrop^® ^ND-1000 UV-vis spectrophotometer (NanoDrop Technologies). First-strand cDNA was synthesized from 1 μg of total RNA template using random hexamers according to the high capacity cDNA archive kit (Applied Biosystems).

### Quantitative PCR

**Figure 1:**
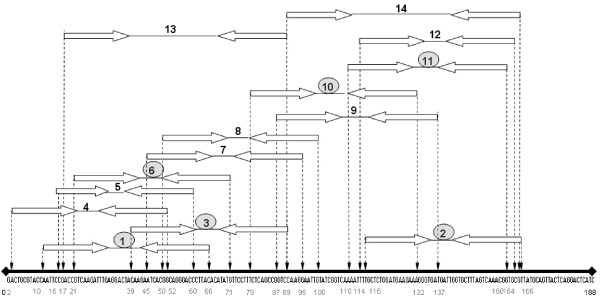
**DNA sequence of the EST [GenBank: **EX956386**] (total length: 188 bp) and binding sites of the 14 PCR primers used in this study**. The primers showing one single product are circled.

We performed Q-PCR assays targeting two expressed sequence tags (ESTs), both presumed to represent single-copy genes. The first EST [GenBank: EX956386] was initially taken from a previously constructed cDNA-AFLP library. The cDNA used to generate the AFLP profile was reverse-transcribed using SuperScript™ III Reverse Transcriptase and was the result of a normalized pool of cDNA from 16 oysters (full-sib families) challenged with high temperature and bacterial infection (Taris, unpublished data). This fragment was cloned into a pCR4-TOPO vector using the TOPO TA Cloning kit (Invitrogen). Sixteen clones were directly sequenced using an ABI 3730XL (Applied Biosystems) automated sequencing system and Big Dye Terminator 3.1 chemistry (Applied Biosystems). The second EST [GenBank: AJ565694] was directly taken from Genbank. The two fragment lengths were respectively 188 and 402 bp. We used Primer Express^® ^Software v2.0 (Applied Biosystems) to design primers for Q-PCR. This software takes into consideration a variety of parameters, including Tm (melting temperature), primer complementarities, and secondary structure, as well as amplicon size. In total, we designed 14 primer pairs for each EST (Table 1). Primers were first chosen according to appropriate design requirements (primers length from 18 to 26 nucleotides), but we also attempted to distribute the amplicons along the entire lengths of the EST fragments (see figure 1 for an example using EST [GenBank: EX956386]).

**Table 1:** Primer pairs design for each EST.

**a [GenBank: **EX956386**]**						
Primer pairs	Oligonucleotide sequences (5'--3')	Start	Length	Tm	%GC	Amplicon length

f1	CCGACCGTCAAGATTTGAGG	16	20	59	55	51
r1	GTGTAAGGGTCCCTGCCGT	66	19	58	63	
f2	GCTCTGGATGAAGAAAGGGTGA	116	22	59	50	51
r2	AACGCACCGTTTGACTAAAGC	166	21	58	48	
f3	ACAAGAATCACGGCAGGGAC	39	20	59	55	51
r3	GACCGGCTGAGAAAGGAACATAT	89	21	59	52	
f4	ACTGCGTACCAATTCCGACC	2	20	59	55	51
r4	GCCGTGATTCTTGTAGTCCTCAA	52	23	59	48	
f5	CCAATTCCGACCGTCAAGAT	10	20	58	50	51
r5	GGGTCCCTGCCGTGATTC	60	18	60	67	
f6	CGTCAAGATTTGAGGACTACAAGAAT	21	26	58	38	51
r6	CATATGTGTAAGGGTCCCTGCC	71	22	59	55	
f7	ATCACGGCAGGGACCCTTA	45	19	59	58	51
r7	TCCTTGGACCGGCTGAGA	95	18	59	61	
f8	GGCAGGGACCCTTACACATATG	50	22	59	55	51
r8	ACAATTCCTTGGACCGGCT	100	19	58	53	
f9	GTCCAAGGAATTGTATCGGTCAA	87	23	59	43	51
r9	TCACCCTTTCTTCATCCAGAGC	137	22	59	50	
f10	CTCAGCCGGTCCAAGGAAT	79	19	59	58	54
r10	CTTTCTTCATCCAGAGCAAAATTTT	132	25	58	32	
f11	AATTTTGCTCTGGATGAAGAAAGG	110	24	59	38	51
r11	CCGTTTGACTAAAGCACCAATCA	160	23	60	43	
f12	TTGCTCTGGATGAAGAAAGGGT	114	22	59	45	51
r12	CGCACCGTTTGACTAAAGCAC	164	21	59	52	
f13	CGACCGTCAAGATTTGAGGACTA	17	23	59	48	73
r13	GACCGGCTGAGAAAGGAACA	89	20	59	55	
f14	CCAAGGAATTGTATCGGTCAAAA	89	23	59	39	77
r14	ACGCACCGTTTGACTAAAGCA	165	21	59	48	

**b [GenBank: **AJ565694**]**						

Primer pairs	Oligonucleotide sequences (5'--3')	Start	Length	Tm	%GC	Amplicon length

f1	CCGGATATGGGACAAACAAATC	148	22	60	45	67
r1	TGGAAACGATGTCGGCTATG	215	20	60	50	
f2	CCTTCATGTATGGGTCCCAAA	169	21	58	48	51
r2	ACTCTGGAAACGATGTCGGC	219	20	59	55	
f3	GTTCCGCAAGGACTATCTCGA	22	21	58	52	51
r3	AAGCTCCCACAGAATTTATTGATGT	72	25	58	36	
f4	ACTCCAAGGCATGTAGCATCG	313	21	59	52	51
r4	TTGGATGACTGTGCC CTTAAAAT	363	23	59	39	
f5	TAACATCAATAAATTCTGTGGGAGCT	46	27	59	33	51
r5	AATTTTCGTGCATCTTTCTCTGC	96	23	59	39	
f6	TGGGACAAACAAATCCTTCATG	155	22	58	41	51
r6	GTCGGCTATGACCTGATTTGG	205	21	58	52	
f7	CCATGATTCGAACACATTGGTG	241	22	60	45	51
r7	ATGCATGATTCCTTGGCCTTA	291	21	58	43	
f8	CCAAGGCATGTAGCATCGCT	316	20	60	55	51
r8	TTTTTGGATGACTGTGCCCTTA	366	22	58	41	
f9	AATTCTGTGGGAGCTTTGCAG	57	22	60	45	51
r9	TCTTTCCTAATAATTTTCGTGCATCTT	107	27	59	30	
f10	ATGGGTCCCAAATCAGGTCA	178	20	59	50	51
r10	GTGGAAGCAACTCTGGAAACG	228	21	58	52	
f11	GCAAGAAAACCGGATATGGGA	139	21	60	48	51
r11	TTTGGGACCCATACATGAAGG	189	21	58	48	
f12	CATGATTCGAACACATTGGTGG	242	22	60	45	51
r12	GATGCATGATTCCTTGGCCT	292	20	59	50	
f13	TATGGGTCCCAAATCAGGTCA	177	21	59	48	51
r13	TGGAAGCAACTCTGGAAACGAT	227	22	60	45	
f14	CGTTTCCAGAGTTGCTTCCAC	208	21	58	52	51
r14	AATGTGTTCGAATCATGGTCGTT	258	23	59	39	

For primer testing, we pooled equal cDNA sub-samples from individual oysters from each family (6 individuals/family) and used 10 ng of this pooled cDNA in each Q-PCR reaction. For each pool, Q-PCR assays were performed in triplicate using SYBR^® ^Green PCR Master Mix (Applied Biosystems) in 25 μl reactions containing cDNA (diluted in 5 μl) and 50 nM (final concentration) of each primer. Each Q-PCR reaction plate included a non-template negative control to ensure the absence of contamination and the data was normalized using *Elongation factor 1 α *[GenBank: AB122066] as the reference housekeeping gene. The consistency of *Elongation factor 1 α *expression was initially evaluated by testing the differences in Ct value within families and triplicates (two-way analysis of variance; Proc GLM [17]). For each plate, no family or replicate effect was shown to be significant (P > 0.05). For both reference and target genes, PCR cycling conditions were: 50°C for 2 min (AmpErase^® ^UNG activation), 95°C for 10 min (AmpliTaq Gold^® ^DNA polymerase activation), 50 cycles of 95°C for 15 s and 60°C for 1 min, and finally, 95°C for 15 min, 60°C for 15 s. The reactions were run and results analyzed using Applied Biosystems 7500 Real Time PCR system (software version 1.4) using the absolute quantification program and included a post-PCR melt curve analysis, to detect nonspecific amplification in cDNA samples. Quantification of gene expression was based on the determination of threshold cycle (*C*_T_-value), defined as the first cycle number with detectable fluorescence above background. The *C*_T _value for each sample was estimated using the automatic baseline setting. Relative quantification was accomplished by normalizing raw C_T _values to the reference gene expressed as *target*/*reference *ratios [ratio = E target^(*C*T target)^/E reference^(*C*T reference)^] where E represents the empirically determined efficiency estimated for each reaction using LinRegPCR software [18]. Options selected to fit the window-of-linearity were a number of data points between five and six and the best correlation coefficient.

### Statistical analyses

The level of cDNA (relative to the reference gene) was analyzed for significant differences between families using Proc GLM [17]. The model was as follows:

*Y*_*ij *_= μ+ fam_*i *_+ rep_*j *_+ ε_*ij*_

where *Y*_*ij *_is the dependant variable (Ct values), μ is the overall mean, rep_*j *_the replicate effect nested with family, fam_*i *_is the family effect and ε_*ij *_the residual error. The analysis of variance was followed by Tukey's multiple comparison procedure whenever a family effect was significant. Significance was assumed for *P *< 0.05.

## Results

**Figure 2:**
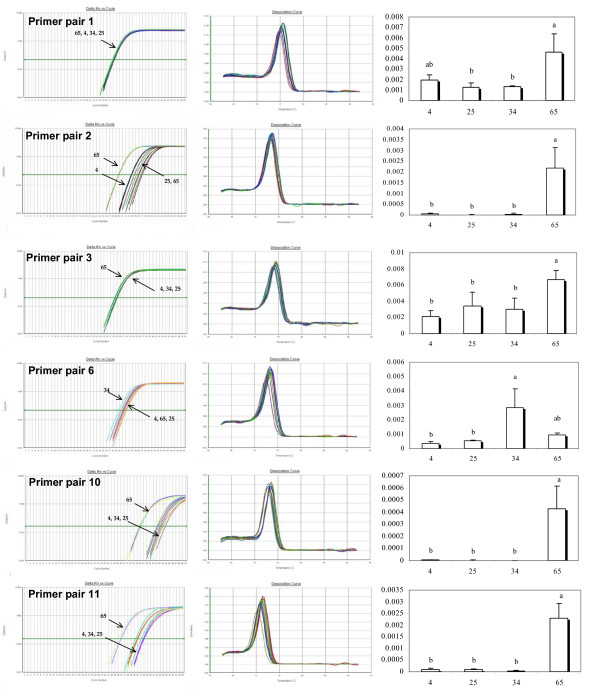
**Outcomes of level of cDNA expression across families for primers (1, 2, 3, 6, 10, 11) technically validated through the analysis of the melting curve of the EST [**EX956386**]**. From left to right: Plot of PCR cycle number against logarithm PCR product amount; Derivative Melting Curve; Statistical results of level of cDNA expression across families based on a multiple comparison test (Tukey). Family numbers are reported on the horizontal axis, histograms sharing a letter are not significantly different (P < 0.05).

**Figure 3:**
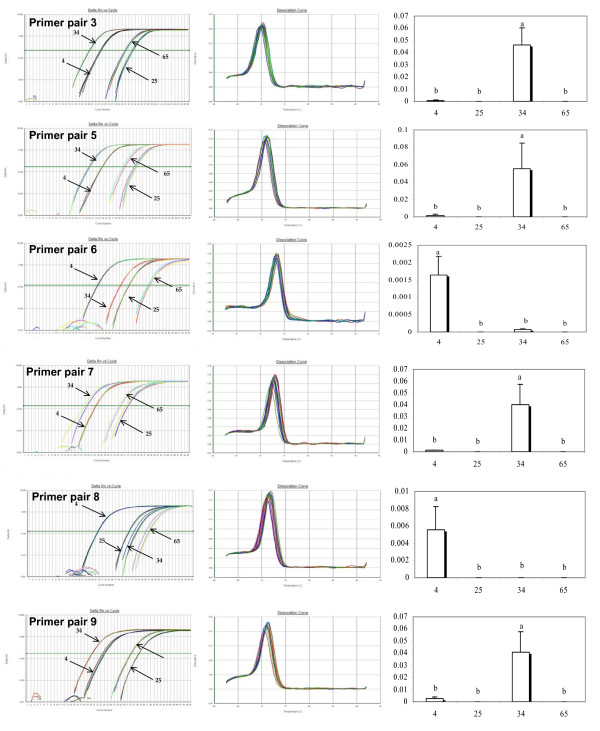
**Outcomes of level of cDNA expression across families for primers (3, 5, 6, 7, 8, 9) technically validated through the analysis of the melting curve of the EST [**AJ565694**]**. From left to right: Plot of PCR cycle number against logarithm PCR product amount; Derivative Melting Curve; Statistical results of level of cDNA expression across families based on a multiple comparison test (Tukey). Family numbers are reported on the horizontal axis, histograms sharing a letter are not significantly different (P < 0.05).

**Figure 4:**
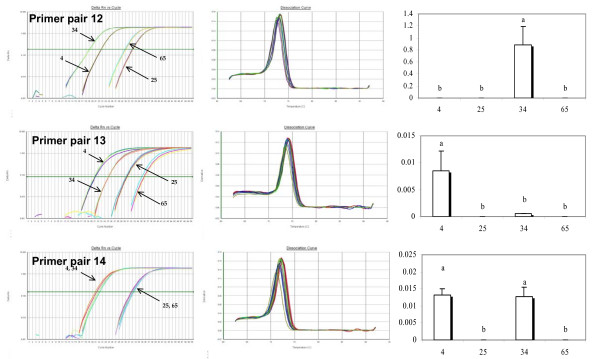
**Outcomes of level of cDNA expression across families for primers (12, 13, 14) technically validated through the analysis of the melting curve of the EST [**AJ565694**]**. From left to right: Plot of PCR cycle number against logarithm PCR product amount; Derivative Melting Curve; Statistical results of level of cDNA expression across families based on a multiple comparison test (Tukey). Family numbers are reported on the horizontal axis, histograms sharing a letter are not significantly different (P < 0.05).

Out of the 14 primer pairs tested per EST, 6 and 9 for [EX956386] and [AJ565694] respectively showed a single product in the melt curve analysis and were thus considered to be worth further consideration and testing. All primer pairs that produced multiple products were eliminated from further consideration. Melt curve analyses, raw data, and statistical outcomes are summarized in figures 2, 3 and 4. We found statistically significant family effects for all primer pairs used, but no significant variation among technical replicates. To more closely examine these significant family effects, we used Tukey's range test to perform multiple comparisons of the four families studied (Table 2).

**Table 2:** Ct values across families and primer pairs, obtained from the ABI 7500 real-time PCR instrument

**a [GenBank: **EX956386**]**								
	Model effect		Family classification test (Tukey)					
				Family				
					
Primer pair		Pr > F	Pattern	4	25	34	65	Pattern

1	Family	0.02	*Ct mean*	28.03	28.24	28.25	27.67	
	Replicate	0.68	*SD*	0.07	0.11	0.07	0.03	
			*Statistical grouping*	ab	b	b	a	A
2	Family	< 0.01	*Ct mean*	33.72	36.49	35.29	29.82	
	Replicate	0.42	*SD*	0.10	0.60	0.74	0.07	
			*Statistical grouping*	b	b	b	a	A
3	Family	0.01	*Ct mean*	28.90	28.90	29.01	28.29	
	Replicate	0.24	*SD*	0.10	0.15	0.03	0.06	
			*Statistical grouping*	b	b	b	a	A
6	Family	< 0.01	*Ct mean*	31.26	31.84	31.51	30.51	
	Replicate	0.26	*SD*	0.18	0.36	0.66	0.75	
			*Statistical grouping*	b	b	a	ab	B
10	Family	< 0.01	*Ct mean*	41.47	42.18	43.43	35.93	
	Replicate	0.42	*SD*	0.57	0.57	0.37	0.15	
			*Statistical grouping*	b	b	b	a	A
11	Family	< 0.01	*Ct mean*	35.79	37.56	37.14	30.56	
	Replicate	0.33	*SD*	0.38	0.38	0.95	0.49	
			*Statistical grouping*	b	b	b	a	A

The statistical grouping refers to figures 2, 3 and 4, and corresponds to the result of a Tukey test based on level of cDNA expression and not Ct values.								

**b [GenBank: **AJ565694**]**								

3	Family	< 0.01	*Ct mean*	23.50	33.10	19.87	31.11	
	Replicate	0.42	*SD*	0.16	0.19	0.12	0.21	
			*Statistical grouping*	b	b	a	b	A
5	Family	< 0.01	*Ct mean*	23.85	33.71	19.68	30.30	
	Replicate	0.39	*SD*	0.06	0.38	0.37	0.28	
			*Statistical grouping*	b	b	a	b	A
6	Family	< 0.001	*Ct mean*	22.38	31.91	29.36	37.88	
	Replicate	0.39	*SD*	0.12	0.09	0.08	0.29	
			*Statistical grouping*	a	b	b	b	B
7	Family	0.01	*Ct mean*	22.27	31.77	19.34	29.43	
	Replicate	0.41	*SD*	0.06	0.40	0.41	0.38	
			*Statistical grouping*	b	b	a	b	A
8	Family	< 0.001	*Ct mean*	21.68	31.10	33.15	36.81	
	Replicate	0.42	*SD*	0.08	0.09	0.56	0.61	
			*Statistical grouping*	a	b	b	b	B
9	Family	< 0.01	*Ct mean*	23.90	34.44	20.34	32.16	
	Replicate	0.44	*SD*	0.38	0.02	0.04	0.11	
			*Statistical grouping*	b	b	a	b	A
12	Family	< 0.001	*Ct mean*	22.59	32.08	19.15	29.92	
	Replicate	0.42	*SD*	0.02	0.09	0.02	0.18	
			*Statistical grouping*	b	b	a	b	A
13	Family	< 0.01	*Ct mean*	21.05	30.56	25.11	35.64	
	Replicate	0.40	*SD*	0.12	0.20	0.02	0.46	
			*Statistical grouping*	a	b	b	b	B
14	Family	< 0.01	*Ct mean*	21.45	31.96	21.72	32.22	
	Replicate	0.17	*SD*	0.67	0.28	0.02	0.26	
			*Statistical grouping*	a	b	a	b	C

For EST [EX956386], three different statistical outcomes were observed (respectively named A, A' and B). For primer pairs 2, 3, 10 and 11, the level of cDNA (relative to *Elongation factor 1 α *mRNA) was significantly higher for Family 65 than for the three other families (pattern A), which belong to the same statistical group (group b as shown on figure 2). In contrast, primer pair 1 distinguishes Family 65 from families 25 and 34, but not from Family 4, which is not statistically different from families 25 or 34 (pattern A'). The last primer pair (number 6), groups Family 34 with the higher cDNA level, significantly different from Family 4 and 25, but not from 65, which shares a statistical grouping with Family 4 and 25 (pattern B).

For EST [AJ565694], three different statistical outcomes are also observed (respectively named A, B and C). For primer pairs 3, 5, 7, 9 and 12, the estimated level of cDNA is significantly higher in Family 34 and there is no difference between families 4, 25 and 65 (pattern A). Using primer pairs 6, 8 and 13 produces a different pattern in which the level of cDNA is significantly higher for Family 4 whereas the level of gene expression in families 25, 34 and 65 are statistically indistinguishable (pattern B). Finally, primer pair 14 produces a third outcome in which families 25 and 65 show significantly lower amounts of gene transcript than families 4 and 34 (pattern C).

**Figure 5:**
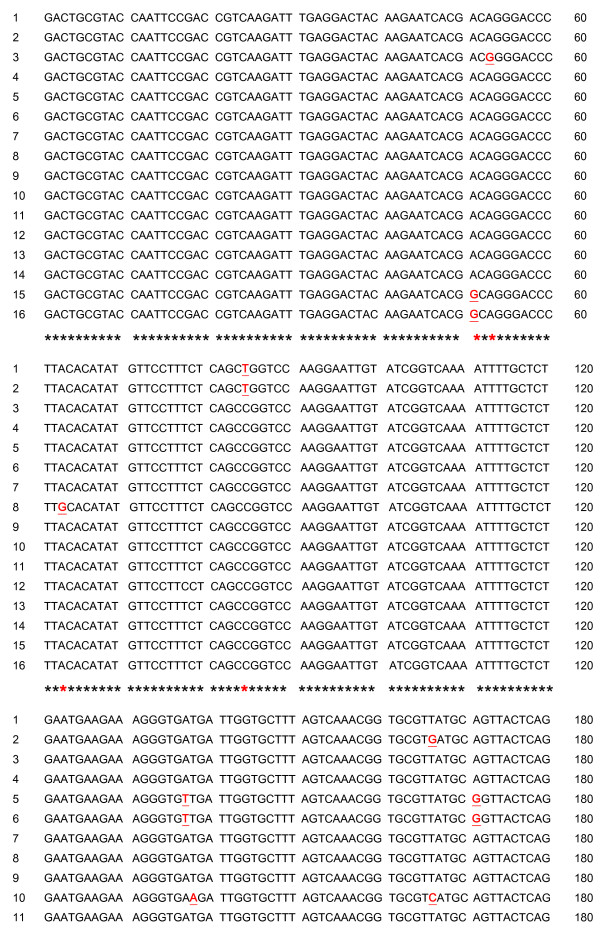
**Sequence alignments of the 16 clones of the 188 bp fragment [GenBank: **EX956386**] initially taken from a cDNA-AFLP library (unpublished data)**. Underlined and red letters flag polymorphism.

As shown in figure 5, after sequencing 16 clones of the 188 bp fragment [EX956386], eight nucleotides appear to be polymorphic, respectively in position 51, 53, 63, 85, 137, 138, 166 and 171.

## Discussion

The variation in expression patterns among families that we observed for the same EST fragment using different primer pairs highlights the complexity of interpreting Q-PCR results and raises serious questions regarding the use of Q-PCR to validate the results of whole-transcriptome screening procedures such as cDNA-AFLP. For both ESTs, depending on the primer pairs used, statistical comparisons of the estimated levels of gene transcription across the four families leads to three different statistical outcomes with different biological implications. Using standard criteria, all of the primer pairs selected would be acceptable insofar as they all produce a single product according to the melting curve analysis. However, different statistical results are obtained with different primers, and it is impossible with these data alone to determine which of these outcomes, if any, is correct.

To address this question more rigorously, it is necessary to look more closely at the plot of PCR cycle number against PCR product amount (figure 2) and the resulting values of Ct (table 2).

Focusing first on EST [EX956386], it is interesting to observe the similarity of Ct values (28 ± 0.5) across families for primer pair 1 and 3 (table 2). The profiles generated by these two primer pairs are distinguishable from those generated by primer pair 2, 10 and 11, but even so the final outcomes show significantly higher level of cDNA expression for Family 65 compared to the other families. For primer pairs 2, 10 and 11, the mean Ct values of Family 65 are respectively 29.82, 35.93, and 30.56, but the mean Ct value of the three other families are at least 4 cycles greater. We hypothesize that the presence of null alleles (i.e. poor primer binding) for Family 4, 25 and 34 but only for primer pair 2, 10 and 11 explains these results

To test this hypothesis, we sequenced 16 clones of the original fragment from the original cDNA-AFLP library. This cDNA is the result of a normalized pool of cDNA collected from 16 individual oysters. An examination of the 16 sequences underlines the presence of polymorphism (figure 5). We observed five of the eight SNPs in more than one clone, making it unlikely, although not entirely impossible, that they include amplification enzyme errors. The polymorphism observed is notably located in the priming site of primers 2, 10 and 11 (figure 1) but also potentially affect the priming site of primer 1, 3 and 6 as well. The case of primer pair 6 is more difficult to interpret. Ct values are close across families. However, the level of cDNA appears to be higher in Family 34. As mentioned before, variation in PCR efficiencies must be accounted for and the raw Ct values cannot be compared directly unless it can be assumed that all PCR reactions had equal efficiencies. This underscores the importance of directly estimating PCR efficiencies because this correction can have substantial impacts on the estimates obtained. In this regard, the use of the Log (fluorescence) versus cycle number plot in the linear regression approach [18] can be viewed as a reliable measure of PCR efficiency. In contrast to the method of serial dilutions based solely on Ct estimates, LinRegPCR analyzes the kinetics of individual Q-PCR reactions and includes a number of data points belonging to the log-linear phase of the PCR reaction (i.e. the exponential phase). Moreover, the method of dilution series results in only one value of efficiency for all dilutions, even though efficiency varies as the input concentration changes [19].

Overall, primer pairs 1 and 3 seem to be unaffected by the observed polymorphisms while primer pairs 2, 10 and 11 under-estimate the level of expression of Families 4, 25 and 34 relative to Family 65 due to null alleles caused by sequence variation in the priming regions even though all of these primer pairs produce a single product in the melt-curve analysis and are thus acceptable by standard criteria.

Turning to our second EST, the same reasoning may be applied, although we do not have access to multiple sequences as for [EX956386]. Pattern A is the most frequent, and is produced by primer pairs 3, 5, 7, 9 and 12. By comparing the Ct values displayed in pattern A (table 2), we can note a certain consistency. Values within Family 4 range from 22.27 to 23.85 across primer pairs, from 31.77 to 34.44 for Family 25, from 19.15 to 20.34 for Family 34, and from 29.43 to 32.16 for Family 65. In sharp contrast, primer pairs 6, 8 and 13, produce pattern B, with the Ct values of families 34 and 65 much higher than in pattern A (above 25 for Family 34 and above 35 for Family 65). Once again, it seems reasonable to conclude that null allele issues in families 34 and 65 that depend on the primer pair used have profound impacts on the estimates and result in an underestimation of the level of expression in the affected families. Finally, pattern C (primer pair 14), is intermediate, presumably affected to a lesser extent by the null allele issue. Overall, patterns B and C seem to be driven by artefacts rather than biology. Pattern A is not only the most frequent (5/9) but also the one corresponding to the most logical explanation.

There are few examples of how sequence polymorphism affects Q-PCR results in the published literature, but, in a recent study, Stevenson *et al. *[20] demonstrated how SNPs within a probe-binding region can adversely influence the sensitivity of real time PCR assays. The idea is that the presence of mismatches (SNPs) between a probe and a sequence target will lower the melting temperature. This conclusion was drawn by using probes for detection of herpes simplex virus. In the present case, such a statement might be applicable as well, even though SYBR Green chemistry is known to be sequence-independent. Sequence polymorphism among alleles in the different families influences the efficiency of primer binding and therefore the overall efficiency of the assays.

## Conclusion

Our study demonstrates that careful and rigorous primer optimization and an examination of sequence variation among families or individuals is a critical step before real time PCR assays are used to complement whole transcriptome analyses, especially when dealing with short fragments such as those generated by differential display techniques. Statistical outcomes can be profoundly influenced by polymorphisms in the sequence under study if they cause poor binding of primers or poor amplification. These artefacts cannot be detected using standard melt-curve analyses because they have purely quantitative rather than qualitative effects. For this reason, it is strongly recommended when working with genetically diverse biological material, to test multiple primers and, if at all possible, to examine the sequences investigated for polymorphisms in priming regions to avoid erroneous conclusions.

## Authors' contributions

NT participated in study design, carried out the laboratory analyses and drafted the manuscript. RPL participated in the study design and sample collection. MDC participated in study design and was involved in the manuscript preparation. All authors read and approved the final manuscript.
